# Effect of Halita mouthwash on oral halitosis treatment: A randomized triple-blind clinical trial

**Published:** 2019-04-24

**Authors:** Zahra Jamali, Mahdieh Alipour, Syamand Ebrahimi, Marzie Aghazadeh

**Affiliations:** ^1^Department of Oral Medicine, Faculty of Dentistry, Tabriz University of Medical Sciences, Tabriz, Iran; ^2^Research Assistant, Dental and Periodontal Research Center, Faculty of Dentistry, Tabriz University of Medical Sciences, Tabriz, Iran; ^3^Faculty of Dentistry, Tabriz University of Medical Sciences, Tabriz, Iran

**Keywords:** 0.2% chlorhexidine mouthwash, clinical trial, Halita mouthwash, halitosis, organoleptic score (OLS)

## Abstract

***Background***. Halitosis (oral malodor) is a common problem all over the world and its prevalence has been estimated at 23‒ 50%. Halitosis originates from oral cavity in 85% of patients. This clinical trial was conducted to evaluate the efficacy of the Halita mouthwash in oral halitosis treatment.

***Methods***. Fifty subjects with an organoleptic score of >2 at baseline participated in this triple-blinded clinical trial. Subjects were divided into 2 groups. Group I subjects (N=25) were instructed to rinse with 0.2% chlorhexidine (CHX) mouthwash twice a day for 1 week. Group II subjects (N=25) used Halita mouthwash with the same instruction. Halitosis was evaluated at baseline and one week after using the mouthwashes by organoleptic method. Data were analyzed with chi-squared and Mann-Whitney U tests (P<0.05).

***Results***. In the Halita group subjects exhibited 2.04±0.65 reduction in OLS. OLS reduction in the chlorhexidine group was 1.95±0.74. Statistical analysis showed no significant difference between the two groups (P>0.05).

***Conclusion***. Based on the results, Halita mouthwash has the same effect on oral halitosis as routine 0.2% CHX mouthwash. Halita mouthwash has fewer side effects because of lower concentration of chlorhexidine. Therefore 0.2% CHX mouthwash could be replaced by Halita mouthwash for the treatment of halitosis.

## Introduction

Halitosis or oral malodor is bad or foul breath, which is very common in the general population and has negative effects on the individual’s quality of life.^1-3^ 80‒90% of halitosis origins’ were in the oral cavity.^4,5^

Volatile sulphur compounds (VSCs) which are involved in halitosis are produced by gram-negative anaerobic oral bacteria.^6^ VSC substrates for bacteria were cysteine and methionine, which are found in saliva, gingiva, cervical fluid and tongue coating debris.^7^

There are four available methods in halitosis measurement: organoleptic measurement method, gas chromatography, sulphide monitoring and the BANA test.^8^ The organoleptic method (OLS) is the gold standard method in halitosis detection. In this method, the exhaled air is smelled by a clinician. Despite its shortcomings, OLS is a reliable, inexpensive, practical and easy method to assess halitosis.^9,10^ Successful halitosis treatment depends on detection of the etiologic agent and implementation of cause-related therapy.^11,12^

1. When the causes are intraoral and related to microorganisms, the treatment approach is:
2. Mechanical reduction, including use of scaling and root planing in a dental office and brushing and flossing at home
3. Chemical method, including the use of mouthwashes
4. Converting volatile fragrant gasses to non-volatile components

Masking of the malodor which is an easier and economical treatment for halitosis by improving oral hygiene with toothbrushing and use of dental floss^13^

Overall, simple treatments such as antibacterial agents are very effective in controlling oral halitosis.^12,14^

Although 0.2% chlorhexidine is considered a routine and effective antiseptic agent, it has side effects such as tooth and tongue staining and taste sensation reduction.,^15,16^ Halita mouthwash contains 0.05% chlorhexidine, 0.05% cetylpyridinium chloride (CPC) and zinc. Due to the lower concentration of chlorhexidine in Halita mouthwash and fewer side effects, this clinical trial was designed to evaluate and compare the efficacy of Halita and 0.2% CHX mouthwashes in the treatment of halitosis.

## Methods

This clinical trial was a randomized, triple-blind study. According to a previous similar study, sample size was estimated at 46.^17^ By considering the loss of samples, 50 subjects (25 males and 25 females) were selected from the population of patients referring to the Department of Oral Medicine, Faculty of Dentistry, Tabriz University of Medical Sciences (TUOMS). All the participants signed informed written consent forms.

### Inclusion Criteria

1. Organoleptic score was >2 at baseline.
2. The participants were 18‒35 years of age.
3. The subjects had no systemic disease and were not taking antibiotics or receiving other antimicrobial therapy.
4. The subjects did not receive the same time treatment for their halitosis.

### Exclusion Criteria

1. Smokers, alcoholics and drug addicts.
2. Patients with periodontitis or pocket depth >6 mm.
3. Patients taking drugs which induced xerostomia.
4. Subjects consuming spicy food: garlic or onions two days before examination.
5. Patients with orthodontic appliances or removable dentures.

The subjects were randomly assigned to one of the following groups by Randlist 1/2 software program and determination of 4-6 blocks based on age and sex; then a code was given to each subject.

**Group I:** The subjects used 0.2% CHX mouthwash for 1 week (twice a day, 10 mL for 40 seconds).

**Group II:** The subjects used Halita mouthwash for 1 week (twice a day, 10 mL for 40 seconds).

The clinician and analyzer used the codes and were blinded to the type of mouthwash the participants used. The subjects, either, did not know the type of mouthwash they used. The participants did not clean or rinse their mouth 6 hours before measuring halitosis; perfumes were avoided, too. At least 2 hours before the examination the subjects did not have any food or liquid and did not use chewing gums.

The gold standard method for halitosis measurement is the organoleptic scoring method (OLS).

In this method, a well-trained clinician smells the exhaled air. Three usual methods in OLS are:

1. Mouth odor smelled at 10 cm from the oral cavity while the patient normally breathes or while the patient counts loudly to 10. This method was used in the current study.
2. Interdental floss (after flossing with dental tape, the odor of the floss is scored).
3. Nasal odor; while the patient is breathing through the nose (closed mouth) the exhaled air is scored.

In the organoleptic method, the clinician gives a score to the intensity of malodor and determines whether malodor exists or not. The score range is 0‒5 which as presented in Table 1.^18^

In this study, one clinician who was blinded to group allocation of the subjects determined organoleptic scores. The participants’ mouth was smelled at baseline and 7 days later. The subjects used the mouthwashes for 7 days. 0.2% CHX mouthwash and Halita mouthwash were stored in similar bottles (with an X mark on jars for each group). The volume of the mouthwash in each bottle was 140 mL, which was prepared for 14 doses for consumption in 7 days. Then oral and written description was given to patients: 2 tablespoons twice a day (in the morning after breakfast and at night before bedtime), to rinse for 40 seconds in the mouth. Drinking, eating and mouth washing should be avoided for one hour after mouthwash use. The mechanical mouth cleaning method was calibrated in two groups using the same toothbrush, toothpaste and brushing method. The subjects used toothbrush and dental floss twice a day, before using the mouthwash. Then the participants in both groups were followed for 7 days and the organoleptic score was measured by the same clinician.

**Table 1:** Organoleptic scoring scale

Rosenberg & McCulloch scale	Description
1 2 3 4 5	No detectable odor Hardly detectable odor Light odor Moderate odor Strong odor Extremely strong odor

Adapted from Rosenberg and McCulloch^18^

### Statistical Analysis

Statistical analysis was performed using SPSS 17. Statistical comparisons of the groups were conducted using ANOVA and Mann Whitney U test. In this study, P<0.05 was considered statistically significant.

## Results

### Characteristics and Oral status of the Subjects

**Figure 1:**
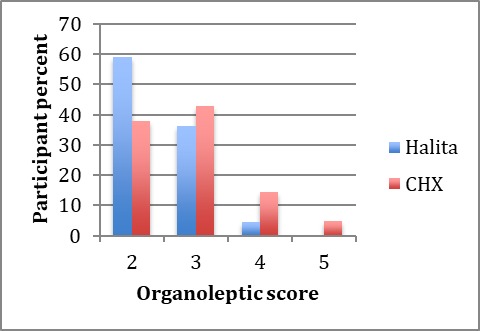
Baseline organoleptic scores in the Halita and 0.2% chlorhexidine groups.

All the 50 subjects completed the study. The mean ages of subjects in the Halita and CHX groups were 23.5 and 23.8 years, respectively. Mann-Whitney U test did not show any significant difference between the ages of the two groups. The baseline organoleptic scores are shown in Figure 1. Chi-squared test did not show a significant difference in OLS at baseline between the two groups (P>0.05).

**Figure 2:**
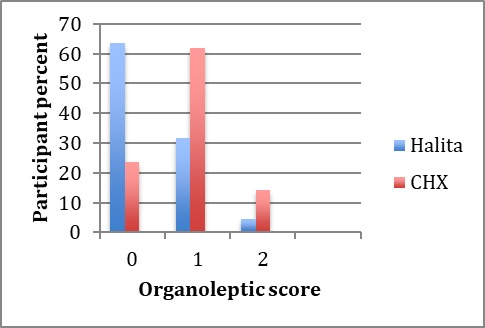
Post-treatment organoleptic scores in the Halita and 0.2% chlorhexidine groups.

Seven days after using mouthwashes, OLS decreased in both groups. The post-treatment organoleptic scores are shown in Figure 2.

**Figure 3:**
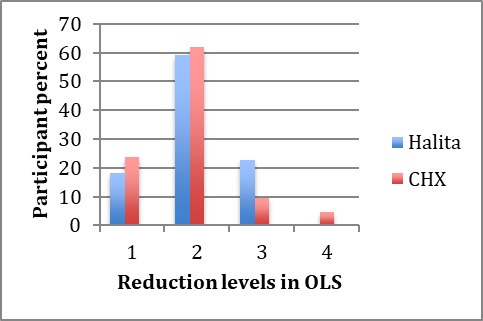
The presence of the participants due to decreases in halitosis scores.

The mean score reduction (mean ± SD) in the 0.2% CHX group was 1.95±0.74, with 2.04±0.65 in the Halita group. The presence of the participants due to decreases in organoleptic scores is shown in Figure 3.

Chi-squared test showed no significant differences in OLS changes between the Halita and 0.2% CHX groups (P>0.05).

## Discussion

Because of great use of mouthwashes and their various formulations and lack of scientific evidence to support their efficacy in different oral problems, this study was conducted to compare the effect of Halita and 0.2% CHX mouthwashes on the treatment of halitosis. The organoleptic scoring scale was used to measure halitosis intensity in this study.

To ensure the right randomization we used Randlist 1/2 software and for triple-blinding codes given to the subjects. In the present study, we uses definite including and excluding criteria, which were used in Kayoko Shinda (2008) study.^7^

Halitosis has intraoral an extraoral origins. Almost 85% of all halitosis cases have an intraoral origin. One of the treatment approaches in malodor problem with intraoral origin is the mechanical and chemical reduction of microorganisms. Mechanical methods (brushing and flossing followed by chemical methods [mouthwashes]) are more effective in halitosis treatment.^19^ This method was used in our study for halitosis treatment. Yadav et al clinical trial in 2015 indicated that 0.2% CHX mouthwash affected tongue coat accumulation, which causes halitosis.^20^ However, this mouthwash has some side effects like irritation of the oral mucosa, greater burning sensation, altered taste perception, changes in the color of composite restorations, brown pigments on teeth surfaces and unpleasant taste and odor in the oral cavity.^15^ Dadamio et al showed that Halita and Meridol mouthwashes were more effective than other mouthwashes in halitosis treatment.^17^

However, in the present study there was no significant difference between Halita and 0.2% CHX mouthwashes for halitosis treatment. Oral hygiene measures were standardized during our study for decreasing bias risk, contrary to a study by Dadamio et al.^17^ Fedorowicz et al^21^ showed that mouthwashes containing 0.05% CHX, 0.05% CPC and zinc were obviously more effective than placebo in reducing VSC compounds that cause halitosis. Zinc ions could capture VSC compounds and reduce halitosis intensity.^22,23^ Halita mouthwash used in our study has zinc ions whereas CHX mouthwash does not contain zinc ions. Lower concentration of chlorhexidine in Halita mouthwash leads to fewer side effects compared to routine 0.2% CHX mouthwash and as shown in this study the efficacy of these two mouthwashes in halitosis treatment was similar. An in vitro study by Aghazadeh et al^24^ evaluated the antimicrobial effects of Halita mouthwash containing chlorhexidine, cetylpyridinium chloride and zinc lactate on *Pseudomonas aeruginosa* and *Staphylococcus aureus*. According to the results of this study, Halita mouthwash had significant effects on reduction of bacterial levels. Tongue could be one of the halitosis origins in the oral cavity. Another laboratory study, recently conducted in Temple University, compared the effects of 12 commercial mouthwashes on a mixture of three bacterial spices frequently isolated from the human tongue dorsum. Perio-Aid, which contains 0.12% chlorhexidine and 0.05% cetylpyridinium chloride, had significantly greater effect on antibacterial activity in vitro.^25^ Sreenivasan et al^26^ demonstrated a significant difference in the antimicrobial effect of CHX and 0.05% cetylpyridinium chloride mouthwash on gram-negative pathogens with fluoride containing mouthwashes.^26^ Gram-negative pathogens are the main source of VSC compounds causing halitosis, and reductions in their amounts can reduce halitosis.^13^ Halita mouthwash contains both chlorhexidine and cetylpyridinium chloride, which could decrease OLS. Our data indicate that OLS reduction exhibited no significant difference between the two mouthwashes. Therefore Halita mouthwash could be used instead of routine 0.2% CHX mouthwash with fewer side effects and the same clinical effects on halitosis treatment.

## Conclusion

It was concluded from the results of the current study that use of Halita and 0.2% CHX mouthwashes resulted in similar effects on the treatment of halitosis. With regard to the low adverse effects of Halita (considering the lower concentration of chlorhexidine), it could be introduced as a suitable mouthwash in patients with halitosis, who complain of adverse effects of CHX mouthwash.

## Acknowledgments

The authors thank all the friends and staff members in the Oral and Medicine Department, who helped us complete the study.

## Authors' contributions

ZJ and MA planned the study. ZJ, MA and MAl performed the literature review. ZJ, MA and MAl performed the experiments and drafted the manuscript. SE performed the experimental procedure. MAl carried out the statistical analyses and interpretation of data. All the authors critically revised the manuscript for intellectual content. All the authors have read and approved the final manuscript.

## Funding

This clinical trial was supported by a research fund from Vice Chancellor for Research (VCR) of Tabriz University of Medical Sciences (TUOMS).

## Competing interests

The authors declare no competing interests with regards to the authorship and/or publication of this article.

## Ethics approval

The Ethics Committee of Tabriz University of Medical Sciences (TUOMS) approved the protocol of this study, which was in compliance with Helsinki Declaration. All the participants signed informed consent forms (Approval No. 939). The trial registration ID in ClinicalTrials.gov registration system was: 2014121520314 N.
